# Indium-Based Micro-Bump Array Fabrication Technology with Added Pre-Reflow Wet Etching and Annealing

**DOI:** 10.3390/ma14216269

**Published:** 2021-10-21

**Authors:** Paweł Kozłowski, Krzysztof Czuba, Krzysztof Chmielewski, Jacek Ratajczak, Joanna Branas, Adam Korczyc, Kazimierz Regiński, Agata Jasik

**Affiliations:** Łukasiewicz Research Network—Institute of Microelectronics and Photonics, al. Lotników 32/46, 02-668 Warsaw, Poland; pawel.kozlowski@imif.lukasiewicz.gov.pl (P.K.); krzysztof.czuba@imif.lukasiewicz.gov.pl (K.C.); krzysztof.chmielewski@imif.lukasiewicz.gov.pl (K.C.); jacek.ratajczak@imif.lukasiewicz.gov.pl (J.R.); joanna.branas@imif.lukasiewicz.gov.pl (J.B.); adam.korczyc@imif.lukasiewicz.gov.pl (A.K.); kazimierz.reginski@imif.lukasiewicz.gov.pl (K.R.)

**Keywords:** infrared photodetectors, thermal evaporation, metallization, wet etching, indium bumps, annealing

## Abstract

Indium-based micro-bump arrays, among other things, are used for the bonding of infrared photodetectors and focal plane arrays. In this paper, several aspects of the fabrication technology of micrometer-sized indium bumps with a smooth surface morphology were investigated. The thermal evaporation of indium has been optimized to achieve ~8 μm-thick layers with a small surface roughness of R_a_ = 11 nm, indicating a high packing density of atoms. This ensures bump uniformity across the sample, as well as prevents oxidation inside the In columns prior to the reflow. A series of experiments to optimize indium bump fabrication technology, including a shear test of single columns, is described. A reliable, repeatable, simple, and quick approach was developed with the pre-etching of indium columns in a 10% HCl solution preceded by annealing at 120 °C in N_2_.

## 1. Introduction

The infrared (IR) detection technology has matured to a point at which it can be used in many advanced imaging applications, such as earth and deep-space observation, environmental and industrial monitoring, etc. The most commonly used IR sensors in such solutions are focal plane arrays, which require a densely packed array of micro-solder bumps to form micro-connections to a readout integrated circuit [1]. A flip-chip method is used for the bonding process [2]. The development of this technology for infrared detectors and focal plane arrays faces many challenges, as the need for higher-resolution devices with ever smaller pixel pitches arises [3]. Despite its long history, there are still various aspects of the technology that can be improved or simplified depending on the specific application. This holds especially true for the fabrication of indium bump arrays, where the reliability, repeatability, homogeneity, simplicity, and cost-effectiveness are the most important issues.

Indium is widely used for electrical interconnections in various semiconductor devices due to its physicochemical properties. This is especially true for the production of infrared photodetectors and focal plane arrays. A high ductility, even at low temperatures, predisposes it as a material of choice for micro-connections in cooled devices [4,5,6], regardless of whether or not it is subjected to thermal cycling due to either cryogenic or thermoelectric cooling. Furthermore, it can reduce the stress resulting from the different values of the thermal expansion coefficients of the detector and the transducer system [7]. Nowadays, in infrared detection applications, typical indium bumps have a diameter between 5 μm and 30 μm. Such short connections provide excellent electrical characteristics (e.g., smaller inductance), which is important for operation at very high frequencies [8,9]. The use of a dense packing of micrometer-sized indium bumps significantly increases the signal-to-noise ratio of the device, and can also decrease noise [10,11]. In addition, the low melting point of indium (156 °C) and high plasticity guarantees no structural damage to both the detector and mount/readout electronics [12,13].

The indium bump array is most commonly fabricated using a lift-off process in conjunction with UV photolithography. There are two methods used for indium deposition, namely electroplating and evaporation. The former is usually the preferred method due to its simplicity and low cost [14]. On the other hand, the latter allows for a better control of the deposition process, as well as achieving better uniformity, especially across large samples. After indium columns are fabricated, a reflow process is performed to form bumps. The most crucial step at this stage is oxide removal, as it can prevent the proper melting of indium. This can result in shape non-uniformity, as well as mechanical and electrical problems during and after the flip-chip bonding. Usually, the oxide removal is carried out during the reflow process itself by using a flux atmosphere. The two most common gases used are hydrogen and formic acid vapors. The former requires a specialized furnace, and either molecular hydrogen or hydrogen radicals can be used as a reducing agent [15,16,17]. The use of formic acid vapors has much lower hardware requirements and it is often integrated into bonding systems. However, it requires the precise control of reducing conditions, such as acid concentration, humidity, and temperature. If the conditions are not controlled properly, the time required for complete oxide removal can be very long [18]. Dry plasma etching can also be used for indium surface oxide removal [19,20]. Another conventional method is an acid bath prior to the reflow process, e.g., in formic or hydrochloric acid [21,22,23]. This technique is very efficient in oxide removal but requires a proper cleansing, and, depending on the acid, corrosive residues or indium over-etching can be present [24,25]. Hydrochloric acid does not leave any residuals on the surface but etches metallic indium from all directions, which can cause the under-cutting of deposited structures. This, in turn, can result in the falling-off of In columns and bump loss [20]. Greer et al. compared the effectiveness of oxide removal in a HCl bath and plasma [20]. They showed high-resolution indium 3d XPS (X-ray photoemission spectroscopy) spectra for pre- and post-treatment samples. Based on the indium metal peak height, their optimized two-step plasma treatment was comparable to a simple 2 min HCl wet etch. In order to take full advantage of the simplicity of the HCl-based approach, a further improvement is necessary. The concentration of the acid in an aqueous solution and the etching time should be optimized to minimize the damage. Furthermore, this method may be regarded as a cost-effective way to form high-quality indium bumps.

In this paper, several aspects of fabricating uniform indium bumps with a smooth surface are discussed. A series of experiments were carried out at each of the four stages of the processing: photolithography, indium evaporation, indium oxide removal before the reflow, and the reflow process. Few oxide removal methods were considered, with a special focus on the bath in hydrochloric acid. The latter gave the best results, namely the shape and dimensional uniformity of indium bumps, as well as a smooth surface. The problem of under-cutting of indium columns in aqueous HCl was solved by introducing additional annealing prior to the bath. Finally, a repeatable and reliable technology of indium bump formation was developed.

## 2. Experimental Procedure

**Figure 1:**
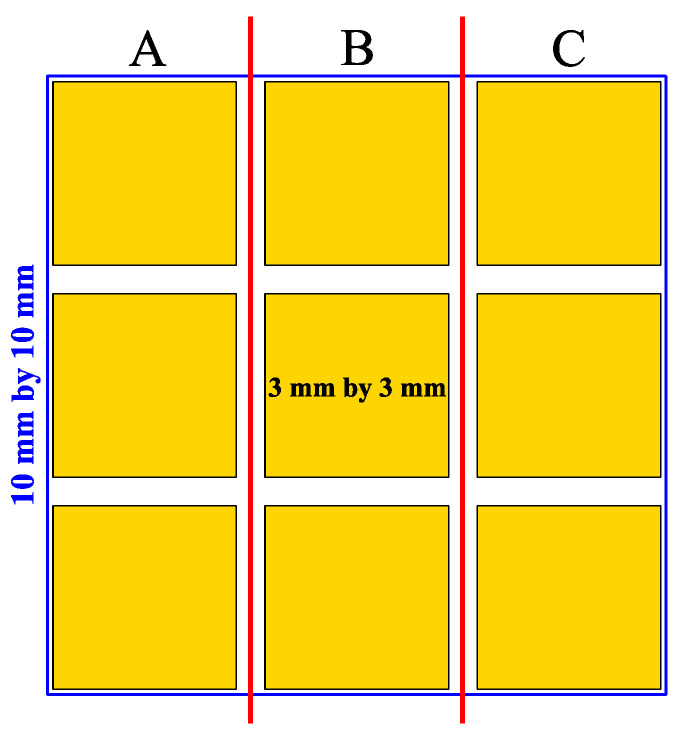
The schematic diagram of samples used in the carried out experiments.

Indium columns were formed using thermal evaporation and a lift-off technique. Epi-ready sapphire substrates with dimensions of 10 mm × 10 mm were used. A single sample consisted of a plate, which was divided into nine squares, each 3 mm × 3 mm. The photolithography masks were designed to allow for creating circular holes in a photoresist with a diameter in the range from 5 μm to 60 μm and with a pitch of 200 μm. In Figure 1, the schematic diagram of a sample is shown. The hole diameters for squares in each column A, B, or C are the same. The main advantage of such an approach was the possibility of choosing various combinations of diameters for holes in the dielectric layer, under bump metallization (UBM), and indium columns. The formation of indium bumps was divided into two stages. The first stage was focused on the UBM made using the knowledge and experience already existing in our group (see Section 2.1). The second stage concerned the formation of spherical indium bumps and was optimized as detailed in the subsequent subsections.

### 2.1. Technology Stage Not Subject to Optimization

The samples for the experiment were prepared through an analogous process to the one described in ref. [6]. During this stage, the AZ^®^nLOF2020 negative photoresist, with 1.5 μm thickness, was used at each step. A gold layer was deposited on sapphire substrates, which served as an optical contrast for the subsequent photolithography processes and contained necessary adjustment marks. Next, a 300 nm-thick SiO_2_ layer was deposited on it at 300 °C using the plasma-enhanced chemical vapor deposition method. The value of the refractive index of the layer was measured to be 1.49 at λ = 632 nm, which is close to the one for stoichiometric SiO_2_. Then, the sample was covered with a negative photoresist, in which, holes with diameters of 5 μm, 10 μm, and 15 μm (columns A, B, and C in Figure 1, respectively) were created. The main role of these holes was to precisely position indium bumps on the sample. Before etching the holes in the SiO_2_ layer, the sample was immersed in an ammonia solution to improve the wetting of the surface by the etchant. Without this step, approximately 30% of the holes were not etched properly. The silicon dioxide was then etched in a buffered HF acid solution, which was prepared and cooled to 16 °C immediately before the start of the process. The diameters of the holes obtained in such a way were 2–3 μm larger than the ones on the photolithography mask. This was attributed to over-etching due to the low adhesion of the SiO_2_ layer to the gold film on the substrate. A thin (few nanometers) Ti layer was deposited on Au to mitigate this issue and improve the adhesion of the dielectric film. Besides titanium, chromium can also be used for this purpose [26]. We decided to use the former due to its compatibility with antimonide-based IR detector technology developed at our institute [27,28]. Next, under bump metallization consisting of titanium, platinum, and gold layers (Ti/Pt/Au) with thicknesses of 10 nm, 20 nm, and 300 nm, respectively, was deposited using magnetron sputtering. The diameters of UBM pads were, 15 μm, 20 μm, and 25 μm in columns A, B, and C, respectively. These templates were used in further experiments.

### 2.2. Optimized Technology Stage

This stage began with indium deposition on the samples prepared in the previous stage. Firstly, holes in the photoresist were prepared for indium evaporation in the photolithography process, which had diameters of 25 μm (column A), 30 μm (column B), and 35 μm (column C). The samples were coated with a 10 μm-thick layer of AZ^®^15nXT 450cPs negative photoresist. Then, the lift-off process was carried out with the use of an ultrasonic bath, which lasted for 10 min. This step was also used as a preliminary test of the attachment of the indium columns to the UBM pads. No indium column loss was observed at this stage. During photolithography, a spray development method was used, which improves the efficiency of the process, especially in the case of thick photoresists. Not completely developed photoresist residuals at the bottom of the holes could cause adhesion-related problems during and after indium deposition. In addition, particular attention was paid to the precise alignment of patterns formed in subsequent photolithography processes.

Prior to the indium deposition, samples were bombarded with argon ions for 3 min (100 W) in Leybold L400 SP sputter coater or with oxygen ions for 30 s in a self-made plasma generator to clear the holes in the photoresist of any residues. The indium was thermally evaporated in the Balzers BA510 machine at a chamber vacuum of 4 × 10^−6^ Torr. The sample was attached to the heat sink made of stainless steel with a diameter of 10 cm and a height of 3 cm. Then, it was cooled down and mounted in the deposition machine. The time between cooling, mounting, and the beginning of chamber evacuation was less than five minutes. The evaporation rate was in the range of 10–60 Å/s and the temperature of the substrate at the beginning of the process was ~10 °C. The quality of the deposited indium was monitored by the surface roughness R_a_ of the indium layer deposited on an epi-ready sapphire substrate with a roughness of less than 10 Å. These control substrates were put in each evaporation process. The surface roughness of indium layers was measured using the KLA Tencor P-16 contact profiler. The evaporation processes were optimized until a mirror-like surface of the In layer was obtained. The smaller the surface roughness, the higher the packing density of In atoms. Such material is more resistant to etching, which results in smaller material loss during oxide removal and improvement of bump uniformity across the sample.

Finally, the reflow of In columns was carried out using Finetech Fineplacer lambda die bonder (Berlin, Germany) at the temperatures (TR) of 160 °C, 180 °C, 185 °C, and 220 °C in the following variations:

(a) Without pre-etching of In columns; reflow in the hydrochloric acid vapors;
(b) Without pre-etching of In columns; reflow in the formic acid vapors;
(c) With wet pre-etching of In columns in formic or hydrochloric acid solution; reflow in the formic acid vapors;
(d) With annealing preceding the wet pre-etching of In columns in hydrochloric acid solution; reflow in the formic acid vapors.

**Figure 2:**
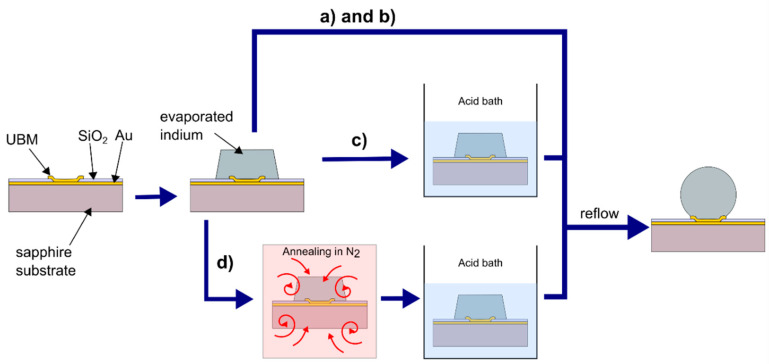
The schematic diagram of the indium bump formation process showing three alternative approaches. (**a**) reflow in the hydrochloric acid vapors without pre-etching of In columns; (**b**) reflow in the formic acid vapors without pre-etching of In columns; (**c**) reflow in the formic acid vapors with wet pre-etching of In columns in formic or hydrochloric acid solution; (**d**) reflow in the formic acid vapors with annealing preceding the wet pre-etching of In columns in hydrochloric acid solution.

In Figure 2, a schematic flow diagram of the aforementioned indium bump formation processes is shown. The conditions and obtained results are summarized in Table 1.

## 3. Results and Discussion

**Figure 3:**
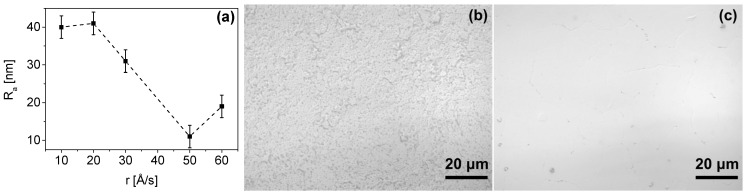
(**a**) Dependence of the roughness R_a_ of the 8 μm-thick In layer as a function of its deposition rate; (**b**) image of the surface of the In layer with roughness R_a_ = 40 nm; (**c**) image of the surface of the In layer with roughness R_a_ = 11 nm.

In Figure 3, the results obtained during the formation of indium columns are summarized. The smallest surface roughness of the indium layer R_a_ = 11 nm was reached for the evaporation rate of 50 Å/s. A further increase in the evaporation rate resulted in the roughening of the In layer due to inefficient heat dissipation through the substrate. Similarly, slower deposition processes resulted in a roughness of 30–40 nm. In Figure 3b,c, the indium surfaces with the highest and smallest roughness are shown, respectively. The best result of R_a_ = 11 nm was obtained when the sample was mounted on a heat sink and cooled before the deposition, as described in the previous section. Observation under an optical microscope revealed a small graininess of evaporated indium columns. This corresponds to a high packing density of In atoms, which inhibits the oxidation of the material inside the column [10]. As a result, a high level of dimensional uniformity, as well as an easier reflow and bonding process, can be achieved. This, in turn, would improve both the electrical and mechanical reliability of micro-connections.

Indium oxidizes very quickly (within a few hours at room temperature) and forms a several nanometers-thick layer [18]. The oxide present on the surface has a much higher melting point (1910 °C) than that of a pure metal (156 °C). The oxide layer hinders the re-melting of indium columns into hyper-hemispherical bumps. The first series of experiments was carried out in hydrochloric acid vapors. Although the obtained results differed in some details, the common features of such a reflow were improperly formed bumps and a cracked surface (samples #1 and #2). This result could be explained by a lack of fine control over conditions in the annealing chamber due to the simplicity of the used setup. It is known that, depending on the water concentration in the atmosphere, the etching rate of indium oxide in HCl vapors can decrease significantly [18].

Formic acid vapor reduces indium oxide much more effectively than hydrochloric acid vapor, but not enough on its own to form proper bumps (sample #3) in a set time. This experiment yielded indium bumps with a good geometry and not bad surface morphology. However, there was a non-uniformity of the shape and height of the bumps throughout the sample. Various HCOOH solution concentrations were tested and none of the indium columns fell off. However, not much difference was observed between each solution. The focus was on achieving both the best morphology and an excellent yield. The experiments so far pointed to the fact that a proper chemical treatment of the indium columns immediately before the reflow process is necessary for the fabrication of high-quality indium bumps. This conclusion was consistent with the results presented in Ref. [20]. In order to reduce the thickness of the indium oxide layer on the surface of the columns, the sample was etched in 10% HCl for 30 s just before starting the reflow. Several tests were carried out at various temperatures (160 °C–220 °C), some of which are shown in Table 1 (samples #4, #5, #6). The best results were obtained at 185 °C—hyper-hemispherical indium bump with smooth morphology (sample #5).

A particularly important issue during the fabrication of indium bumps was whether all of the evaporated indium columns formed bumps after the reflow or not. It was observed that, in the experiments without wet pre-etching, all UBM pads were occupied, even though bumps were not properly formed. This was proved by observation of the samples under an optical microscope after indium deposition and reflow. It was also determined that none of the indium columns fell off during the lift-off process in an ultrasonic bath (10 min, 800 W). This confirmed a good attachment of the evaporated indium to the UBM pads.

**Figure 4:**
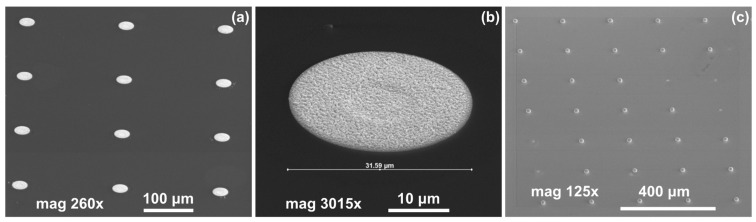
(**a**) SEM images of empty spots after indium bumps; (**b**) an empty spot after indium bump; (**c**) an array of indium bumps with some missing.

In the next step of optimization, samples were first pre-etched in a 10% HCl solution for 20 s, and then a reflow in formic acid vapors was performed at temperatures of 160 °C, 185 °C, and 220 °C for 20 s. The same optical evaluation of samples was made as mentioned above. It was observed that, after the reflow, on average, four to five entire squares (Figure 1) filled with bumps were missing, regardless of the reflow parameters. In Figure 4a,b (SEM images), empty spots left by indium bumps are shown. The image obtained at the highest magnification (Figure 4b) showed a rough surface of a UBM pad, which indicated the presence of In and/or Au-In intermetallic compounds. Otherwise, the pad should be smooth, as the surface of sputtered gold would be mirror-like. The diameter of the field, which was comparable to the base of the indium column, suggested that they fell off either before or during the reflow. The addition of pre-etching in HCl was the only change in the process, and it was determined to be the cause. This is consistent with the work of other researchers, which showed that, despite excellent oxide removal in aqueous HCl, indium columns were prone to falling off due to excessive under-etching.

In Table 2, the changes introduced to several technological steps that were made to resolve the issue of falling-off bumps and their effects are gathered. The default indium bump fabrication technology (row No. 0) described earlier was set as a starting point for further optimization. The changes in each row were applied only one at a time to the default process flow.

In the first step, no ultrasonic bath during the lift-off process was used (row No. 1). We suspected that it could mechanically weaken the interface between the indium column and the UBM pad. As a result, acid could more easily under-etch them, and they could break off due to the violent nature of the reaction. However, no change in yield was observed after pre-etching and reflow, which proved that the ultrasonic bath was not the cause. Next, a 10% solution of HCl during the pre-etching stage was exchanged for a 5% solution in an attempt to decrease the etching rate (row No. 2). This time, there was around a 10% improvement in the average yield. This result was unsatisfactory. Finally, additional annealing prior to the pre-etching was added (row No. 3). The idea behind this change was that the AuIn_2_ intermetallic compound was shown to be the first one to form on the In-Au interface. The thickness of this phase increases with time, even at low temperatures [29]. Increasing the temperature accelerates the process of AuIn_2_ phase formation, which should lead to strengthening the bonding between indium columns/bumps to UBM pads [30]. The proposed annealing was carried out at 120 °C in an N_2_ atmosphere and lasted for 3 min. In contrast to the aforementioned four to five entire squares of indium bumps falling off, the addition of this step resulted in a 100% indium bump yield. This means that there were bumps present in every square on the sample. The yield is understood as no missing bumps after reflow if the indium columns were present after evaporation.

**Figure 5:**
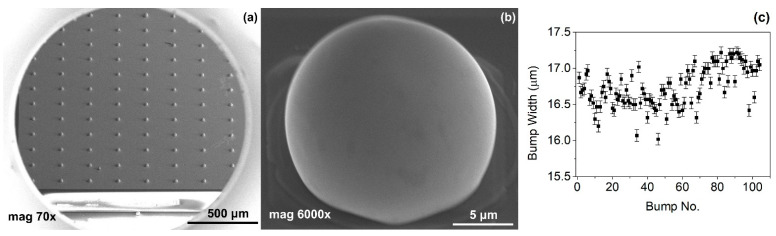
(**a**) A low magnification SEM image of a square with indium bump array; (**b**) a high magnification SEM image of a single bump fabricated using technology with additional annealing; (**c**) error bar graph of bump widths measured using SEM microscopy.

In Figure 5, SEM images of indium bumps fabricated using the approach with annealing and wet etching in HCl before the reflow are shown. A low magnification image of the square with an indium bump array on the sample demonstrating a 100% yield is depicted in Figure 5a. A high magnification image of a single ~17 μm-wide bump is shown in Figure 5b. A smooth surface and good geometry can be observed. In this paper, indium bumps of four sizes were fabricated. In Section 2, it was mentioned that three pairs of UBM and indium column diameters were considered, namely d_UBM_ = 15 μm and d_In_ = 25 μm, d_UBM_ = 20 μm and d_In_ = 30 μm, and d_UBM_ = 25 μm and d_In_ = 35 μm. The indium layer thickness was 8 μm. Based on the truncated sphere theory (TST) the width of the bumps were 20.1 μm, 23.3 μm, and 26.8 μm, respectively [31]. All experiments described thus far were carried out using these bumps. A 100% yield using the proposed approach was achieved for indium bumps of all considered sizes (see an example in Figure 5a). Next, an indium bump with an appropriate size for infrared focal plane arrays application was fabricated. The diameters of the UBM and indium column were chosen as 5 μm and 20 μm, respectively. This combination gave the desired hyper-hemispherical shape. The thickness of the evaporated In layer was 8 μm, as before. The bump width estimated from TST was equal to 16.9 μm. Widths of over a hundred bumps were measured using SEM microscopy. A mean width of 16.7 μm was obtained with a maximum of 17.22 μm, minimum of 16.02 μm, and standard deviation of 0.27. In Figure 5b, an example of such a bump is shown. Good geometry and smooth surface morphology can be observed. The error bar graph with the spread of the bump width is shown in Figure 5c.

To confirm the obtained results, it was checked whether both edge-related effects originating from a spin coating of the emulsion during photolithography and the small sample size had any effect on the obtained results. This could especially cause a problem during evaporation and lift-off of indium columns due to the nonuniformity of the photoresist thickness. For this purpose, the process described in the previous sections was repeated on a substrate 30 mm × 30 mm. The pattern shown in Figure 1 was placed in the middle of the sample. The same situation, in which indium columns fell off during the pre-etching without prior annealing, was observed. The number of missing bumps did not vary much on average between these two experiments.

To further investigate the issue of falling off the evaporated indium columns during the acid bath, a shear test was performed. A self-made setup was based on an Axis FC20 force gauge, which measures forces of up to 20 N with a resolution of 5 mN. The samples were placed on the vacuum chuck mounted on the XYZ-θ stage. The shearing blade was attached to the force gauge, which was mounted on a motorized, precision, single-axis stage driven using a stepper motor. The motion was controlled from a PC with dedicated software via a microcontroller-based interface.

**Figure 6:**
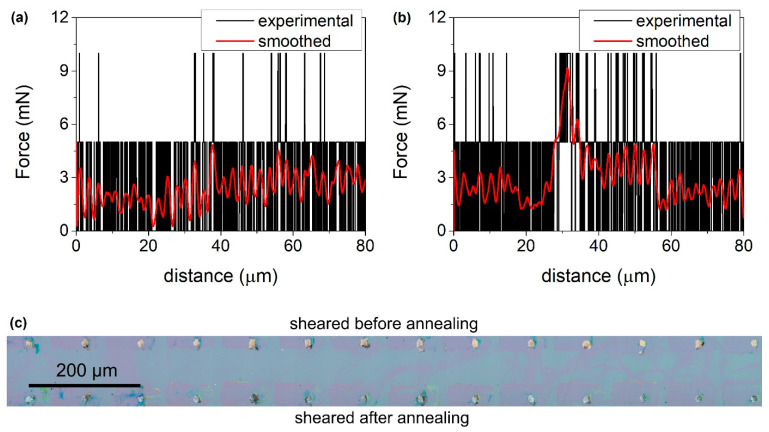
Force versus distance data for 35 μm in diameter indium columns before (**a**) and after (**b**) annealing (black line—experimental data, red line—the result of adjacent-averaging processing). (**c**) Optical microscope image of residues after shearing of indium columns.

The following experiment was performed. Two 20 mm × 20 mm samples were fabricated using the procedure described in Section 2. The shear test was performed on the first sample. In the beginning, the columns with various diameters were sheared to evaluate the force necessary for their removal, as well as the capability of the gauge to measure it. Based on the results, the sensitivity of the setup allowed for the proper evaluation of the largest columns (35 μm in diameter). Thus, 45 of such indium columns from various places on the sample were sheared. In Figure 6a, an example of the measured force (black line) as a function of distance covered by the blade is shown. Signal processing using the adjacent-averaging approach with a weighted average and window of 125 points was performed on the experimental data. These settings were kept constant throughout this experiment. As can be seen, no usable signal was detected and/or extracted. The force required to shear as-evaporated In columns was below the noise of the setup.

After the completion of the previous stage, the sample was annealed according to the procedure given in this paper (see Table 2, row No. 3). Next, another shear test of 45 indium columns was performed on rows adjacent to the ones tested before. In Figure 6b, an example of a measured force signal (black line) is shown. Raw data were also processed, and evident peaks were observed for all tested columns, e.g., in Figure 6b, red line, peak after ~30 μm travel distance. A series of measurements on the areas of the sample, where no columns were present, were performed to ensure that these peaks were caused by the shearing of indium columns. The signal obtained from these test measurements was similar to the one shown in Figure 6a, albeit smaller. An average apparent fracture force of 9.2 mN was obtained.

In addition to the shear test, samples were observed under an optical microscope. Figure 6c shows an image of the aftermath of the shearing of two neighboring rows of In columns. The top row was sheared before and the bottom row was after annealing. In both cases, there are traces of the material on the surface of the sample, which was smeared during the shear test. A color difference was observed between the residues. The one before annealing was goldish, whereas the one after was silverish. This result is in line with the fact mentioned in one of the previous paragraphs (the first paragraph after Table 2), namely, that annealing hastens the growth of the indium-rich AuIn_2_ phase. On the other hand, directly after the evaporation, various Au-In intermetallic compounds should be present, including Au-rich ones, e.g., Au_2_In. The sample after the annealing and shear test was submerged in hydrochloric acid. No indium columns fell off at that time. For control purposes, the second sample also underwent a shear test, and was then etched as the first one. The results of the shear test were the same as the ones obtained for the first sample before annealing. After the acid bath, it was observed that some indium columns fell off. It is also noteworthy that, during these experiments, there were no cases of UBM pads being sheared off; they stayed firmly attached to the substrate. After the analysis of all of the results, it is clear that the proposed annealing procedure improves both the adhesion of evaporated indium to the UBM and the bump yield.

## 4. Conclusions

Several aspects of indium bump fabrication technology were investigated. The thermal indium evaporation was optimized to achieve the smallest surface roughness (R_a_ = 11 nm), indicating a high packing density of In atoms. This was important for further processing, especially at the surface oxide removal and reflow stages. Three main approaches to the reflow were considered: without the pre-etching of In columns—reflow in the acid vapors; with wet pre-etching in the acid bath—reflow in the acid vapors; with annealing preceding the acid bath—reflow in the acid vapors. The best results were obtained for the reflow process in formic acid vapors preceded by consecutive annealing in a N_2_ atmosphere at 120 °C for 3 min and pre-etching in 10% hydrochloric acid for 20 s. Fabricated In bumps had a very good shape and dimensional uniformity across the sample. This method allowed for both the achieving of the highest yield of 100% and the smoothest bump surface. The annealing before the pre-etching solved the problem of indium columns falling off during the HCl acid bath, which improved the repeatability and reliability of the process. A shear test was performed, which showed a clear increase in the apparent fracture force after annealing, which indicates a better adhesion to the UBM. Furthermore, the time of the reflow was very short. Indium bumps with widths of ~17 μm, ~20 μm, ~23 μm, and ~27 μm were successfully fabricated. The developed approach should be applicable for <10 μm-wide bumps. Additional research is necessary to fully explain the role of pre-etch annealing in the yield improvement, as well as to optimize the presented approach by quantifying the mechanical properties of the In bumps. We believe that this technology can be successfully used in the fabrication of the micro-connections in infrared focal plane arrays based on type-II InAs/GaSb superlattices.

## Figures and Tables

**Table 1:** Results of the performed reflow processes.

No.	Vapor	Pre-Etching	T_R_ [°C]	SEM Image	Comments
#1	HCI	no	185	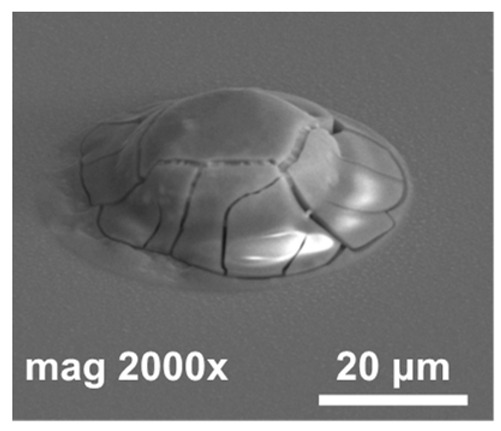	The indium bump was not formed properly. Indium was covered with a cracked layer of indium oxide.
#2			220	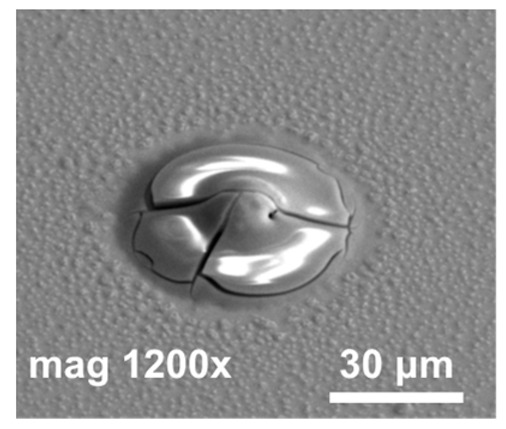	Indium mostly evaporated. The rest was covered by a cracked oxide layer.
#3	HCOOH	no	185	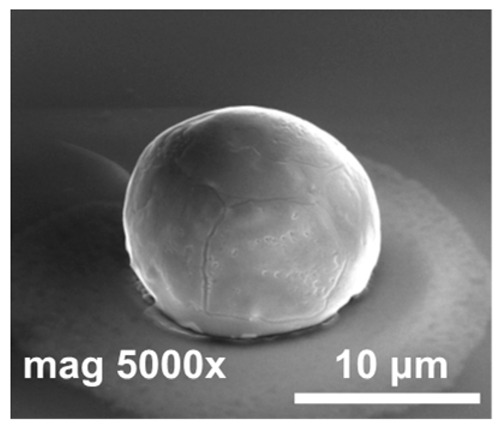	The oxide thickness was significantly smaller than that in #1 and #2 (reflow was carried out within 20 min after removing the sample from the vacuum, in case of #1 and #2—at most 24 h).
#4	HCOOH	10% HCl	160	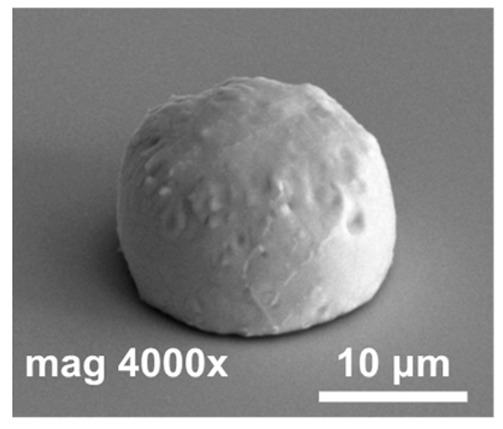	The indium column did not melt down completely, so the bump was not formed properly.
#5			185	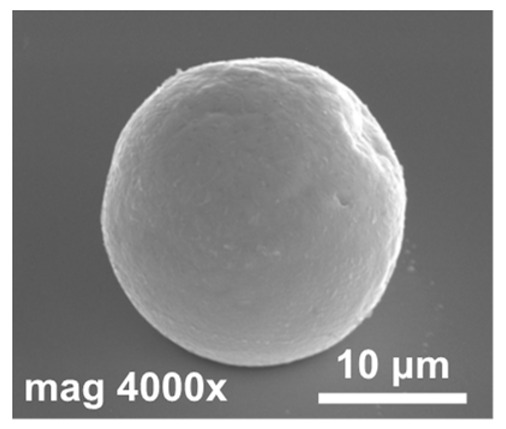	Proper reflow process. A hyper-hemispherical indium bump with a good morphology was obtained.
#6			200	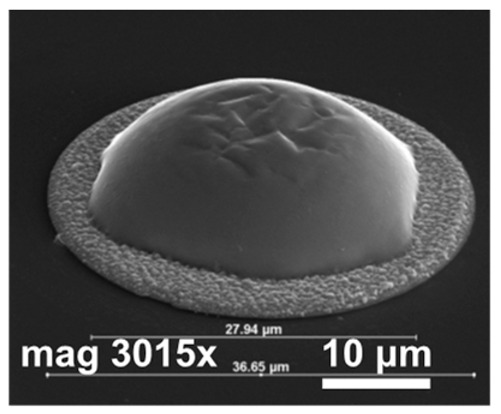	Some of the indium evaporated, and the residue was visible as a ring. The rest melted, but a proper bump was not formed due to a lack of material.

**Table 2:** Optimization of indium bump fabrication with wet pre-etching in hydrochloric acid.

No.	Technological Step	Introduced Change	Avg. Yield [%]
0	default fabrication technology	no	~50
1	lift-off after indium evaporation	no ultrasonic bath	~50
2	pre-etching of In columns	5% HCl (20 s)	~60
3	additional annealing	annealing at 120 °C in nitrogen for 3 min before pre-etching in HCl	100

## Data Availability

Data sharing does not apply to this article.

## References

[1] Rogalski A., Martyniuk P., Kopytko M. (2016). Challenges of small-pixel infrared detectors: A review. Rep. Prog. Phys..

[2] Caccia M. (2001). The challenge of hybridization. Nucl. Instrum. Methods Phys. Res. Sect. A Accel. Spectrometers Detect. Assoc. Equip..

[3] Mailliart O., Renet S., Berger F., Gueugnot A., Bisotto S., Gout S., Mathieu L., Goiran Y., Chaira T. Assembly of very fine pitches Infrared focal plane array with indium micro balls. Proceedings of the 2019 22nd European Microelectronics and Packaging Conference & Exhibition (EMPC).

[4] Huang Y., Lin C., Ye Z.-H., Ding R.-J. (2015). Reflow flip-chip bonding technology for infrared detectors. J. Micromech. Microeng..

[5] Walther M., Schmitz J., Rehm R., Kopta S., Fuchs F., Fleißner J., Cabanski W., Ziegler J. (2005). Growth of InAs/GaSb short-period superlattices for high-resolution mid-wavelength infrared focal plane array detectors. J. Cryst. Growth.

[6] Breibach J., Lübelsmeyer K., Mäsing T., Rente C. (2001). Development of a bump bonding interconnect technology for GaAs pixel detectors. Nucl. Instrum. Methods Phys. Res. Sect. A Accel. Spectrometers Detect. Assoc. Equip..

[7] Huang Q., Xu G., Yuan Y., Cheng X., Luo L. (2010). Development of indium bumping technology through AZ9260 resist electroplating. J. Micromech. Microeng..

[8] Chu K.-M., Lee J.-S., Cho H.S., Park H.-H., Jeon D.Y. A fluxless flip chip bonding for VCSEL arrays using silver coated indium solder bumps. Proceedings of the 2004 International IEEE Conference on the Asian Green Electronics (AGEC).

[9] Kanazawa S., Yamazaki H., Nakanishi Y., Ueda Y., Kobayashi W., Muramoto Y., Ishii H., Sanjoh H. (2017). 214-Gb/s 4-PAM Operation of Flip-Chip Interconnection EADFB Laser Module. J. Light. Technol..

[10] Bah M.A., Manasson A., Outten C., Robinson M., Zhang C., Schumacher J., Desmarais B., Douglass D. (2018). Indium bump deposition for flip-chip micro-array image sensing and display applications. Proc. SPIE.

[11] Jong J.P., Varadaraajan S., Matthews J., Schetzina J.F. (2002). **;** Schetzina, J.F. UV detectors and focal plane array imagers based on AlGaN p-i-n photodiodes. Opto-Electron. Rev..

[12] Jiang J., Tsao S., O’Sullivan T., Razeghi M., Brown G.J. (2004). Fabrication of indium bumps for hybrid infrared focal plane array applications. Infrared Phys. Technol..

[13] Broennimann C., Glaus F., Gobrecht J., Heising S., Horisberger M., Horisberger R., Kästli H., Lehmann J., Rohe T., Streuli S. (2006). Development of an Indium bump bond process for silicon pixel detectors at PSI. Nucl. Instrum. Methods Phys. Res. Sect. A Accel. Spectrometers Detect. Assoc. Equip..

[14] Tian Y., Liu C., Hutt D., Stevens B. Electrodeposition of indium for bump bonding. Proceedings of the 2008 58th Electronic Components and Technology Conference.

[15] Bergh A.A. (1965). Atomic Hydrogen as a Reducing Agent. Bell Syst. Tech. J..

[16] Sabat K.C., Rajput P., Paramguru R.K., Bhoi B., Mishra B.K. (2014). Reduction of Oxide Minerals by Hydrogen Plasma: An Overview. Plasma Chem. Plasma Process..

[17] Furuyama K., Yamanaka K., Higurashi E., Suga T. (2018). Evaluation of hydrogen radical treatment for indium surface oxide removal and analysis of re-oxidation behavior. Jpn. J. Appl. Phys..

[18] Schoeller H., Cho J. (2009). Oxidation and reduction behavior of pure indium. J. Mater. Res..

[19] Huang Y., Lin C., Ye Z.-H., Liao Q.-J., Ding R.-J. (2015). CH4/Ar/H2/SF6 Plasma Etching for Surface Oxide Removal of Indium Bumps. J. Electron. Mater..

[20] Greer F., Dickie M., Vasquez R.P., Jones T.J., Hoenk M.E., Nikzad S. (2009). Plasma treatment methods to improve indium bump bonding via indium oxide removal. J. Vac. Sci. Technol. B Microelectron. Nanometer Struct..

[21] Conti F., Hanss A., Fischer C., Elger G. (2016). Thermogravimetric investigation on the interaction of formic acid with solder joint materials. New J. Chem..

[22] Merken P., John J., Zimmermann L., Van Hoof C. (2003). Technology for very dense hybrid detector arrays using electroplated indium solderbumps. IEEE Trans. Adv. Packag..

[23] Kim J., Schoeller H., Cho J., Park S. (2008). Effect of Oxidation on Indium Solderability. J. Electron. Mater..

[24] Etching Indium to Remove Oxides. https://www.indium.com/blog/etching-indium-to-remove-oxides.php.

[25] Furuyama K., Higurashi E., Suga T. Hydrogen radical treatment of printed indium solder paste for bump formation. Proceedings of the 2017 IEEE CPMT Symposium Japan (ICSJ).

[26] Ruffino F., Grimaldi M.G. (2018). Morphological Characteristics of Au Films Deposited on Ti: A Combined SEM-AFM Study. Coatings.

[27] Czuba K., Sankowska I., Jureńczyk J., Jasik A., Papis-Polakowska E., Kaniewski J. (2017). Influence of Be doping placement in InAs/GaSb superlattice-based absorber on the performance of MWIR photodiodes. Semicond. Sci. Technol..

[28] Smoczyński D., Czuba K., Papis-Polakowska E., Kozłowski P., Ratajczak J., Sankowska I., Jasik A. (2020). The impact of mesa etching method on IR photodetector current-voltage characteristics. Mater. Sci. Semicond. Process..

[29] Deillon L., Hessler T., Hessler-Wyser A., Rappaz M. (2014). Growth of intermetallic compounds in the Au–In system: Experimental study and 1-D modelling. Acta Mater..

[30] Lian J., Chun S.J.W., Goorsky M.S., Wang J. (2009). Mechanical behavior of Au–In intermetallics for low temperature solder diffusion bonding. J. Mater. Sci..

[31] Chiang K.-N., Yuan C.-A. (2001). An overview of solder bump shape prediction algorithms with validations. IEEE Trans. Adv. Packag..

